# Influence of Particle Size on Toughening Mechanisms of Layered Silicates in CFRP

**DOI:** 10.3390/ma13102396

**Published:** 2020-05-22

**Authors:** Julia Hutschreuther, Raphael Kunz, Josef Breu, Volker Altstädt

**Affiliations:** 1 Bavarian Polymer Institute and Department of Polymer Engineering, University of Bayreuth, Universitätsstraße 30, 95447 Bayreuth, Germany; Julia.hutschreuther@uni-bayreuth.de; 2 Bavarian Polymer Institute and Department of Inorganic Chemistry I, University of Bayreuth, Universitätsstraße 30, 95447 Bayreuth, Germany; raphael.kunz@uni-bayreuth.de (R.K.); josef.breu@uni-bayreuth.de (J.B.)

**Keywords:** interlaminar-fracture toughness, layered silicate, carbon-fiber-reinforced composite, prepreg

## Abstract

Carbon-fiber-reinforced epoxies are frequently used for lightweight applications that require high mechanical properties. Still, there is potential regarding the improvement of the interlaminar-fracture toughness. As matrix toughening with nanoparticles is one possibility, in this study two different layered silicates are used to reinforce carbon fiber composites. The first type is a synthetical K-Hectorite (K-Hect) with outstanding lateral extension (6 µm) that has shown high toughening ability in resins in previous work. The other is a commercial montmorillonite (MMT) with a smaller size (400 nm). The aim of this study is to show the influence of the particles on mode I and mode II fracture toughness, especially the influence of particle size. Therefore, double-cantilever-beam tests and end-notched-flexure tests were carried out. Additionally, the fracture mechanisms were investigated via scanning electron microscopy (SEM). It is concluded, that the larger Hectorite particles are beneficial for mode I fracture behavior because of enhanced toughening mechanisms. One the other hand, the mode II energy dissipation rate is increased by the smaller montmorillonite particles due to sufficient interaction with the formation of hackling structures.

## 1. Introduction

Carbon-fiber-reinforced polymers (CFRPs) are a ubiquitous material in modern life. They are light and resilient and provide various possibilities for modification to make the material suitable for many kinds of high-end application. One application where the outstanding properties of CFRPs are needed is in the primary structure of airplanes. In the Airbus A350XBW and the Boeing 787, the fuselage and wings are mostly made of carbon fiber composites. In total, the amount of composites in these airplanes is more than 50% of total weight [1]. Nevertheless, interlaminar failure is still a challenging problem. To improve the interlaminar-fracture toughness and the damage tolerance of the fiber-reinforced composites, efforts have been made to optimize the material toughness. Addressing the reinforcing fibers, there is the possibility of three-dimensional (3D) fiber reinforcement (stitching, Z-pinning or 3D preforms) [2] or surface modification of the fibers [3]. Other approaches that aim to reinforce the resin rich region between the fibers are interleaving [4] or matrix toughening [5,6].

Matrix toughening can be achieved through three different groups of additives: (1) rubber (particles or liquid rubber) [7]; (2) thermoplastics (particles or fibers) [8]; and (3) inorganic particles [9,10]. The first two methods can effectively improve the resin toughness, but suffer from a lowered elastic modulus and glass transition temperature. In contrast, inorganic particles can improve fracture toughness without the deterioration of these properties [11,12]. There is special interest in (platy) inorganic nanoparticles [13] because a low filler content is needed to achieve a high surface area. It has been shown in the past that many different factors influence the toughening of neat resin. The most important ones are the size of particles, the aspect ratio, the compatibility of the filler and the matrix, the dispersion quality and the orientation of particles [14,15]. In earlier work we have shown that a high particle diameter and aspect ratio are beneficial for a number of toughening mechanisms [11,16]. Dispersion quality is also an important factor for maximizing the influence of the inorganic nanofiller [17,18]. Based on these parameters, several toughening mechanisms have been suggested [19,20,21]. Inorganic particles lead to interactions like crack deflection [22], crack pinning [23,24,25,26], crack tip bifurcation [27], microcracking [28] and matrix deformation [29]. These mechanisms are significantly increased when the particles are larger than the crack-tip-opening displacement of the propagating crack. This has been worked out by Kothmann, Ziadeh et al. with large diameter synthetic clay particles with a lateral dimension of > 1 µm [15,30]. In sum, much research has shown that the incorporation of inorganic particles, especially layered silicates, is a successful method for resin toughening with basically clear toughening mechanisms.

**Figure 1:**
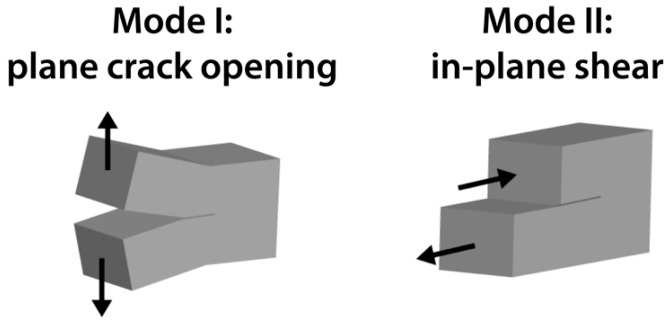
Different loading modes of fracture mechanics.

As a consequence, layered silicates were also used with the intention to improve the interlaminar-fracture toughness of fiber-reinforced composites by matrix toughening. In contrast to neat resin, the mechanisms are more complicated due to the anisotropic material properties and the geometrical restriction of plastic deformation and crack propagation by the fibers. In general, two different loading modes are investigated, the opening mode I and the shear loading mode II. Both are illustrated in Figure 1.

In the case of the mode I load, there is some research stating that fracture toughness—given by the value of energy release rate *G*_Ic_—increases through adding natural clay to the epoxy matrix of fiber composites [27,31]. The authors explain that toughening mechanisms are similar to those in resins, for example crack pinning or crack-tip bifurcation. In contrast to the improvement of fracture toughness in neat resin, the improvement of interlaminar-fracture toughness in laminates is in general lower, as can be nicely seen in the reviews of Liu [6] or Tang [32]. As a possible reason for that behavior, a limitation of plastic deformation by the reinforcing fibers is given [6,33]. There are also publications stating that the addition of layered silicates decreases the energy release rate *G*_Ic_ [33,34,35,36]. Important factors that are discussed in this context are filtering effects leading to a high local clay concentration in the resin rich layer. Therefore, the dispersion quality is low, and agglomerates act as defects rather than tougheners. This idea has been approved by Phonthammachai et al., who have investigated the influence of surface modification of natural clay on *G*_Ic_ [37]. They have shown that the silanization of clays leads to a better compatibility to the epoxy and better dispersion. This results in an increase of *G*_Ic_ with modified clay, while unmodified clay causes a decrease of *G*_Ic_. Subramaniyan et al. add the orientation and the alignment of particles between the plies as possible reasons for nanocracks because the interface represents easy pathways for crack propagation [34]. 

**Figure 2:**
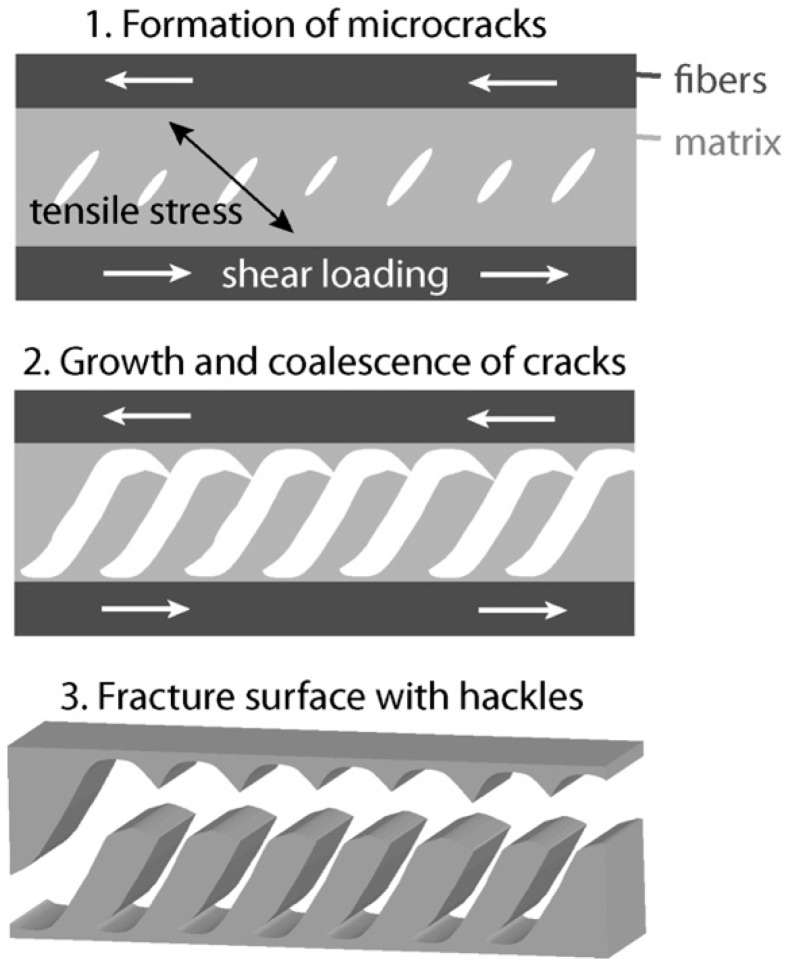
Fracture mechanism in fiber-reinforced composites under mode II loading: hackling.

The influence of clay on the mode II fracture toughness is not related to the behavior under the mode I load. Quaresimin et al. show a clear increase of *G*_IIc_ by the addition of clay, while *G*_Ic_ is decreasing [33]. On the other hand, Subramaniyan et al. show a decrease in both *G*_Ic_ and *G*_IIc_ [34]. The differences can be explained by the change of fracture mechanism. While the crack is propagating more or less in one plane in mode I and is just deflected by particles, there is a special mechanism in mode II, which is illustrated in Figure 2. The external shear forces cause tensile stresses within the resin rich regions between the fibers. Microcracks are formed and grow until the coalescence of the cracks leads to a catastrophic failure. The formed structures are called hackles [38]. Subramaniyan et al. mention that the orientation of particles or a decrease of fiber–matrix adhesion through the clay could be the reason for the decrease of *G*_IIc_ [34]. But in general, there is low information in the literature about the influence of clay on *G*_IIc_ and the fracture mechanisms of CFRP.

For both *G*_Ic_ and *G*_IIc_ there are additional factors that have to be considered when comparing the results of different publications. First, the fiber types differ. Besides carbon fibers, glass fibers are assessed in some publications. Further, for same fiber type, surface modifications can seriously change the fiber–matrix adhesion, and therefore the overall fracture behavior of the fiber-reinforced composites. An example is given by Quaresimin et al. [36]. They could not detect a significant influence of clay on the energy release rate in laminates, while a toughening effect was seen in neat resin. The reason was found in a very low fiber–matrix adhesion that led to early failure, before the toughening effects of clay were observed. Furthermore, the architecture of fibers varies between unidirectional (UD) and bidirectional (woven) reinforcements. While UD reinforcement enables fiber bridging during mode I testing, this effect is less pronounced with bidirectional reinforcement. The fiber volume content additionally influences the effects of fillers on interlaminar-fracture toughness. While Siddiqui et al. more than doubled *G*_Ic_ with the addition of up to 7 wt % of clay in a laminate with 20% fiber volume content [27], the improvement was much lower in materials with higher fiber volume content from 50–70% [31,34,37]. This is because of a higher restriction of toughening effects and plastic deformation with higher fiber content. Other factors that influence the interlaminar-fracture toughness in fiber-reinforced composites are the resin system, processing method and conditions or specimen geometry [39].

This shows that a variety of factors influence the interlaminar-fracture toughness. Therefore, it is important to investigate single effects to gain deeper understanding of the overall system step by step. In this work, the influence of two different layered silicates on *G*_Ic_ and *G*_IIc_ of unidirectional reinforced CFRP are investigated. The fillers are a natural MMT with an average lateral extension of 400 nm and a synthetical K-Hect with an average lateral extension of 6 µm. Therefore, the main focus of this study lies in the effect of particle size on the interlaminar-fracture toughness and the mechanisms of fracture.

## 2. Materials and Methods

### 2.1. Materials and Sample Preparation

The used resin diglycidyl ether of bisphenol A (DGEBA) DER 331 was obtained from Olin (Clayton, MO, USA) and the hardener 4,4’-Diaminodiphenyl sulfone (4,4’-DDS) Aradur 9664-1 from Huntsman (The Woodlands, TX, USA). 

The organic modifier was synthesized by mixing 10,000 g of triphenylphosphine (38 mmol) from Alfa Aesar (Haverhill, MA, USA) and 11.826 g of N-(6-bromohexyl) phthalimide (38 mmol) from TCI (Tokyo, Japan) with 500 mL of toluene from Merck (Darmstadt, Germany) and refluxing the solution for 2 days. The solvent was then removed by distillation and the crude product purified by crystallization from acetone (Merck). (Yield: 87%) 1H-NMR (300 MHz, CDCl3): 7.84 (d, 4H); 7.35 (m, 15H); 3.57 (t, 2H); 2.46 (m, 2H); 1.70 (m, 2H); 1.29 (m, 6H). 

Two different clays were used. The synthetic sodium fluorohectorite with the chemical formula Na_0.5_[Mg_2.5_Li_0.5_]Si_4_O_10_F_2_ and a cation exchange capacity of 110 meq/100 g was synthesized through melt synthesis according to a published procedure [40]. Therefore, a synthetic glass precursor (Na_2_Li_2_Si_6_O_14_) and a mixture of magnesium oxide and silicon dioxide as well as magnesium fluoride were heated in an open glassy carbon crucible up to 1265 °C and quenched after reaction. In order to increase the aspect ratio of the tactoids, the Na^+^ interlayer ions of the pristine material were exchanged for Mg^2+^ to create a more shear-labile state by increased swelling. This material was then applied to shear forces in a stirred media mill to end up with a tuned aspect ratio of roughly 500 and an average lateral extension of 6 µm. Subsequently, another cation exchange for K^+^ lead to a shear-stiff nano mica (K-Hect) with no intracrystalline reactivity [41,42,43]. Hydrophilic bentonite (Montmorillonit clay, MMT) from Merck with an average lateral extension of 400 nm was used as commercial benchmark material. Both nanofillers were organically modified with the same synthesized phosphonium cation to ensure a good compatibility with the resin matrix. The phosphonium bromide dissolved in ethanol (1 mg/ml) was added to a 1 wt % suspension of the respective nanofiller in a ratio determined by streaming potential measurements (Stabisizer®, Particle Metrix GmbH, Inning am Ammersee, Germany). The organoclays were washed with both ethanol and water by centrifugation. A flaky filler material was obtained by freeze-drying a suspension in water with 1 wt % solid content. 

The resin was mixed with clay to obtain a masterbatch with 9 wt % clay content. This mixture was treated in a three-roll mill (EXAKT 80, EXAKT Advanced Technologies, Norderstedt, Germany) to disperse the particles homogenously without the addition of solvents. The masterbatch was then diluted with further neat resin to get suspensions with contents of a layered silicate of 1, 2, 3, 6 and 9 wt % for epoxy-clay-nanocomposites and 2, 4 and 6 wt % for CFRP, respectively. They were heated up to 110 °C, and 4,4’-DDS hardener was added stoichiometrically in respect to the resin content, and the mixture was stirred for 20 minutes. The suspensions were directly used for the production of resin plates or frozen for later use in prepreg production. For resin plates, the suspensions were degassed under vacuum for 10 minutes and subsequently cured in an aluminum mold for 2 h at 140 °C and 2 h at 200 °C. Afterwards, a diamond blade saw was used to fabricate samples for mechanical characterization.

For laminate production, the prepared resin solutions were heated to 40 °C before processing on a prepreg line. A resin film was built on a silicone carrier paper. The high tensile carbon fiber HTS 40 (12 K) from TohoTenax/Teijin (Chiyoda, Japan) was used for fiber reinforcement. The fibers were impregnated with the resin using a calender to produce a prepreg with a width of 20 cm and an aerial weight of 136 ± 2 g/m^2^. Prepregs were cut into pieces and 36 layers were stacked by hand lamination to a unidirectional (UD) laminate. A teflon film was inserted between the two center layers to act as crack initiator for mechanical testing. This laminate was cured in an autoclave process with the following temperature profile: 0.5 h 80 °C, 0.5 h 100 °C, 1.5 h 120 °C, and 2 h 180 °C. Again, a diamond blade saw was used to fabricate samples for different characterization methods.

### 2.2. Characterization 

The filler and the fiber content of all samples were determined via thermogravimetric analysis (TGA) using a TG 209 F1 Libra (Netzsch Gerätebau GmbH, Selb, Germany). The nanocomposites were heated up to 1000 °C under synthetic air flow with a heating rate of 10 °C/min. The CFRPs were first dried at 120 °C for 2 h, then heated up to 450 °C (isotherm of 4.5 h) to determine the fiber content, and afterwards heated up to 1000 °C to determine the remaining clay content. 

**Figure 3:**
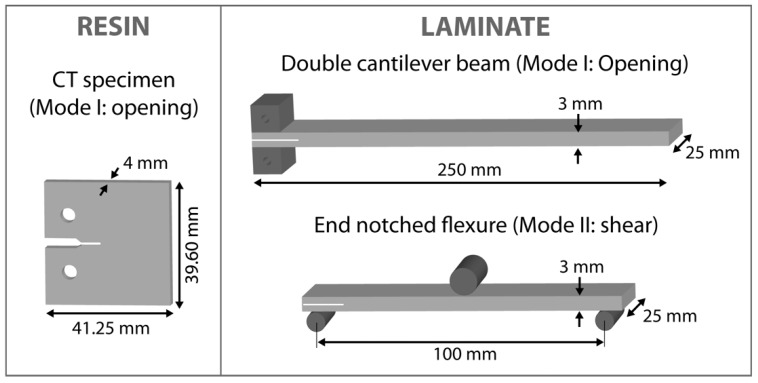
Geometries of specimen for evaluation of fracture toughness in resin and laminates.

The fracture toughness of nanocomposites was determined according to ISO 13586 at a universal testing machine (Zwick Z2,5, ZwickRoell GmbH & Co. KG, Ulm, Germany). Compact tension (CT) specimens were used. A sharp crack was initiated by tapping a razorblade into the machined V-notch. Figure 3 displays the specimen’s dimensions. The stress intensity factor *K*_Ic_ and energy release rate *G*_Ic_ were evaluated by the following equations.

(1) $$K_{\mathrm{Ic}}=\frac{F_{\max}}{d\cdot \sqrt{w}}\cdot f\left(\frac{a}{w}\right)$$

where *F*_max_ is the maximum force required for crack propagation, *d* the thickness of the specimen, *a* is the initial crack length, *w* the specimen width, and *f(a/w)* is a geometrical term defined in ISO 13586.

(2) $$G_{\mathrm{Ic}}=\frac{K_{\mathrm{Ic}}{}^{2}}{E}$$

where *E* is the Young’s modulus. The values were calculated as an average of at least five samples for each material.

Prior to the mechanical characterization of the laminates, ultrasonic tests were performed in order to ensure that the quality of the laminates was good. The interlaminar-fracture toughness of CFRPs was tested in mode I (*G*_Ic_) according to DIN EN 6033 with a double-cantilever beam (DCB) as well as in mode II (*G*_IIc_) according to DIN EN 6034 with an end-notched-flexure (ENF) test. The specimens are shown in Figure 3. In both cases at least five samples were tested for each material. Therefore, laminates were cut in fiber direction into samples that were 25 mm wide and 250 mm long with a thickness of 3 mm. A teflon film inserted in the middle layer during hand lay-up served as the initial crack. Two aluminum blocks were glued onto the edges of the DCB. The blocks were used to apply a tensile load that opened the sample. The measured value was the energy release rate *G*_Ic_, which was calculated by Equation (3):

(3) $$G_{\mathrm{Ic}}=\frac{A}{a\cdot w}$$

where *A* is the energy to achieve the total propagated crack length, *a* is the propagated crack length, and *w* is the width of the specimen.

Afterwards, the DCB sample was cut to get a new initial crack length of 40 mm. This sample was used for the determination of the energy release rate *G*_IIc._ The setup of a three-point bending test with a distance between supports of 100 mm was used to apply shear forces in the cracked laminate. *G*_IIc_ was calculated by Equation (4):

(4) $$G_{\mathrm{IIc}}=\frac{9\cdot P\cdot a^{2}\cdot d\cdot 1000}{2\cdot w\left(1/4\cdot L^{3}+3a^{3}\right)}$$

where *d* is the crosshead displacement at crack delamination onset, *P* is the critical load to start the crack, *a* is the initial crack length, *w* is the width of the specimen, and *L* is the span length.

An optical analysis of the fracture surface of resin and CFRP with and without layered silicates was carried out using scanning electron microscopy (SEM; Zeiss 1530 FESEM).

## 3. Results and Discussion

### 3.1. Filler and Fiber Contents

**Figure 4:**
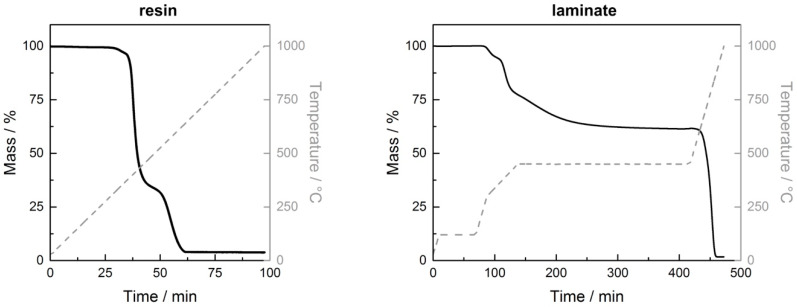
Representative curves of thermogravimetric analysis of resin (left) and laminate (right) for illustration of evaluation of filler and fiber content (here: samples with 6 wt % of K-Hect in neat resin).

Thermogravimetric analysis was used to determine the filler and fiber contents. Figure 4 shows representative curves for the evaluation of layered silicate content in neat resin (left) as well as the evaluation of layered silicate and carbon fiber content in laminates (right). The filler weight content can be determined at the plateau at the end of the curve. In laminates, two plateaus were detected. In the region of the first one, resin was decomposed and the left mass represented the combination of fibers and fillers. After further heating, fibers were also decomposed, so that only the layered silicates were left. By means of material densities (resin+hardener: 1.19 g/cm^3^, carbon fibers: 1.77 g/cm^3^ and layered silicates: 2.7 g/cm^3^), the filler volume content and fiber volume content were calculated. In resin, the maximum MMT and K-Hect content were 2.2 vol % 2.5 vol %, respectively. The laminates showed a fiber volume content of 52 ± 3 vol %. Therefore, the overall filler contents in laminates with maximum 1.0 vol % MMT and 1.1 vol % K-Hect were roughly half the values in resin.

### 3.2. Properties of Epoxy-Clay-Nanocomposites

**Figure 5:**
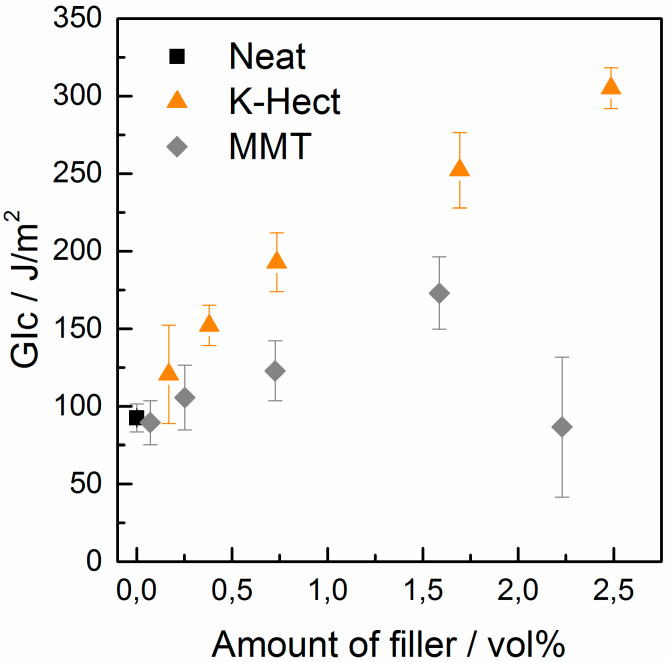
Energy release rate of diglycidyl ether of bisphenol A (DGEBA) nanocomposites with different amounts of montmorillonite (MMT) or K-Hect.

The incorporation of clay into epoxy resin results in an increase in the dissipated fracture energy (Figure 5). The increase of the energy release rate was almost linear for K-Hect and led to a maximum increase of 229% from 93 J/m^2^ in neat resin to 305 J/m^2^ in nanocomposite with 2.5 vol % of K-Hect. There was a lower increase with MMT up to a filler content of 1.6 vol % to a maximum increase of 87%. Further increase of MMT content could not improve the fracture toughness but led to a severe decrease in *G*_Ic_.

**Figure 6:**
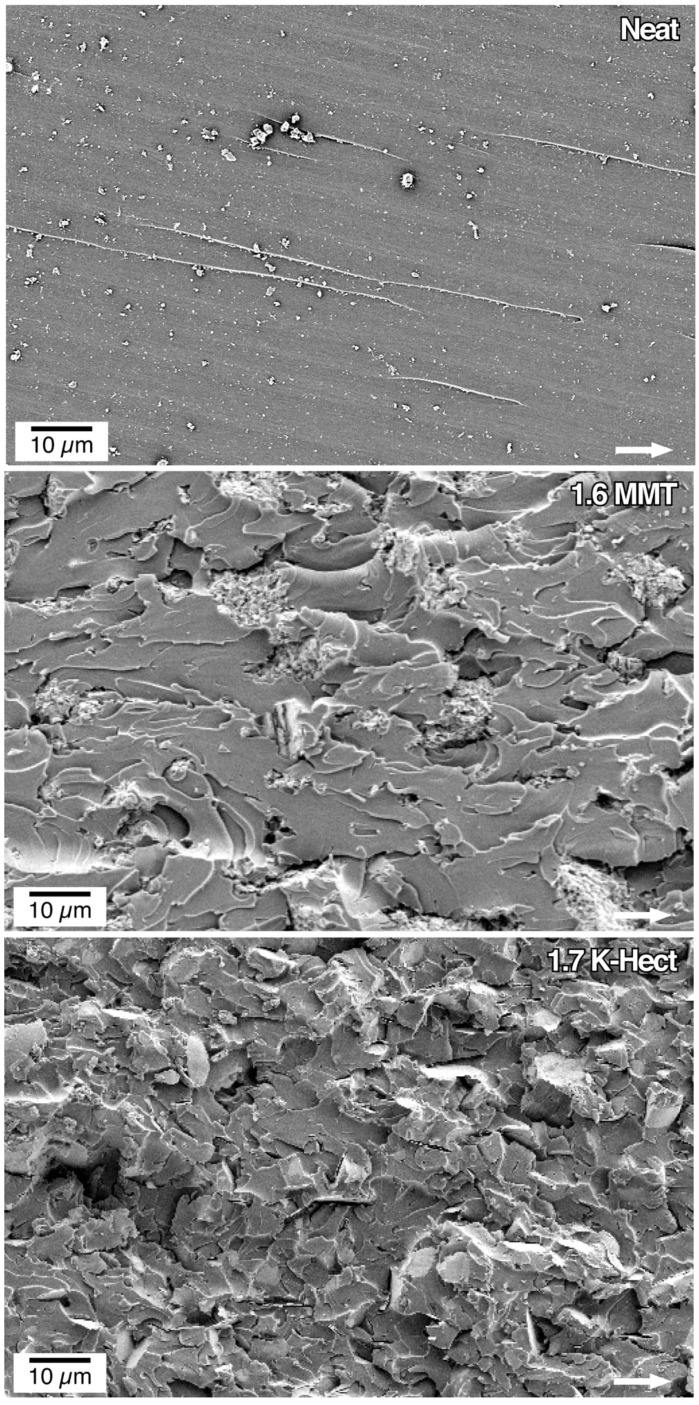
SEM pictures of the fracture surface of compact tension samples (filler content in vol % is given in the pictures). The white arrow shows the direction of crack propagation.

SEM images help to obtain more insight into fracture mechanisms (Figure 6). The neat resin showed a smooth fracture surface and no plastic deformation, indicating brittle fracture behavior and low energy release rate *G*_Ic_. Samples with MMT as well as K-Hect particles had an increased roughness. The larger surface area correlated with an increase of the energy release rate *G*_Ic_ with increasing clay content (Figure 5). The increase in *G*_Ic_ was based on an elongation of crack path initiated by both MMT and K-Hect particles. The MMT particles had a relatively low size of 400 nm. As stated by Kothmann et al. [15], these particles are smaller than the plastic zone that has a diameter in the range of 2 µm for the used DGEBA-44’-DDS system. Therefore, the crack path will not be changed seriously. The increase of the fracture toughness and the roughness of the surface will mainly be caused by particle debonding and the formation of voids. As can be seen in Figure 6, the particles were not fully dispersed, but agglomerates can be found. These agglomerates increased with higher particle content. Large agglomerates in the region of crack path can act as defects, which leads to a decreased fracture toughness. As the agglomerates were not spread uniformly in the samples, the standard deviation was very high for 2.2 vol % of MMT. Similar limitation of the increase in fracture toughness with higher filler content can be found in other clay toughened systems [14]. For the samples with K-Hect, this limitation cannot be seen up to the maximum content of 2.5 vol %. As can be seen in Figure 6, the platelets were homogenously distributed and did not build agglomerates. The reason for this observation can be a more homogenous surface modification of K-Hect due to the homogenous charge distribution of the synthetical layered silicate. Additionally, shear forces in the three-roll mill will more likely separate larger particles. The roughness of the surface of K-Hect toughened resin was much higher than with MMT due to additional toughening mechanisms. With 6 µm, the particles were larger than the plastic zone and could effectively elongate the crack path by crack deflection. Crack bridging and platelet pullout further increased the dissipated energy during crack propagation. In summary, the large K-Hect particles provided more toughening mechanisms and therefore higher fracture toughness. This is in accordance with former investigations [15].

### 3.3. Properties of CFRP

**Figure 7:**
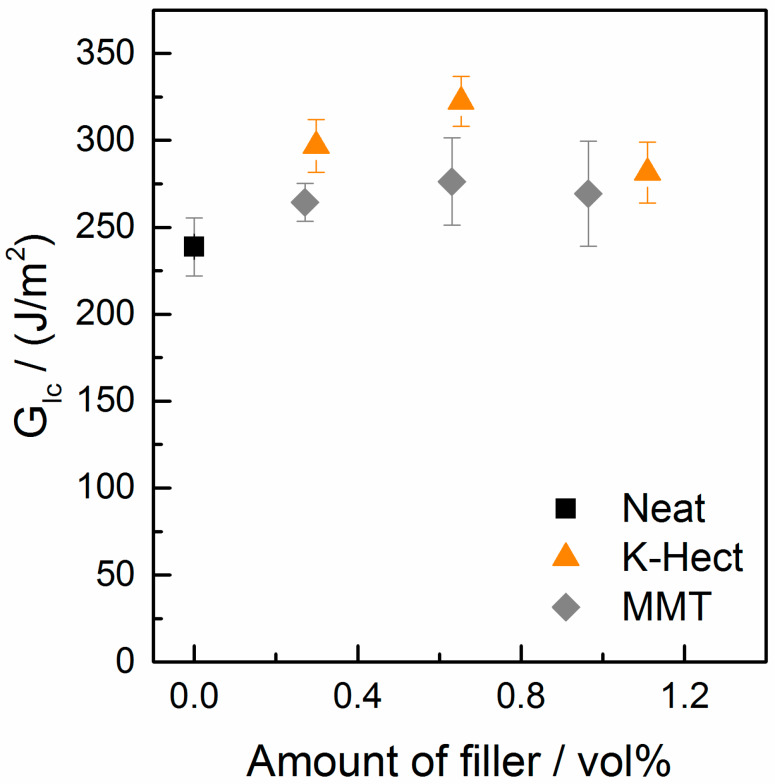
Energy release rate of mode I of laminates with different amounts of MMT or K-Hect.

**Figure 8:**
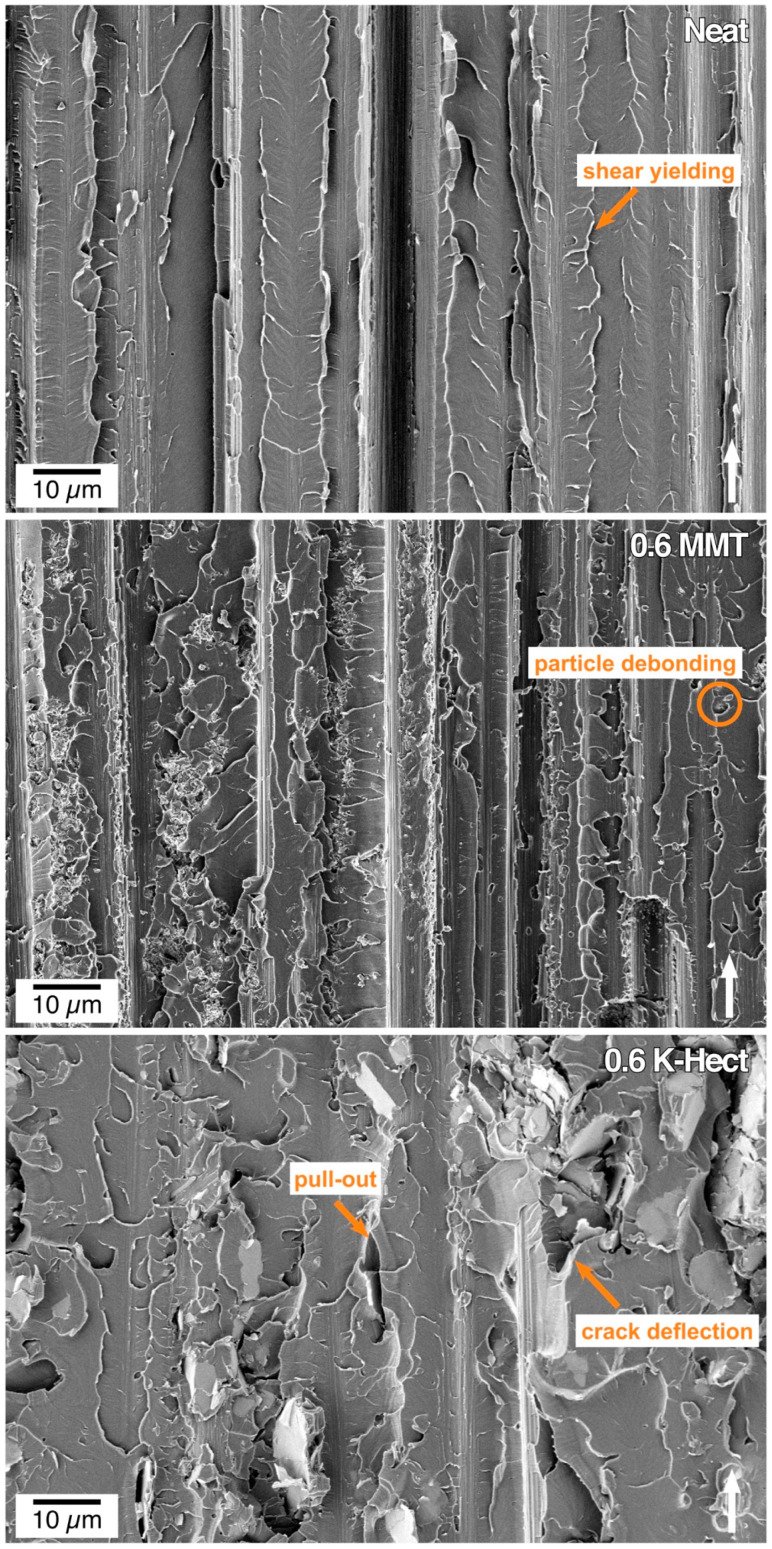
SEM pictures of fracture surface of double cantilever beam samples (filler content in vol % is given in pictures). The white arrow shows the direction of crack propagation.

After proving that natural as well as synthetic clay particles were suitable for toughening epoxy resins, the resin-clay-suspensions were used as matrix in CFRP. It should be investigated whether the high increase in fracture toughness can also be seen in laminates. As shown in Figure 7, for the CFRP with K-Hect particles, the energy release rate *G*_Ic_ increased by 35% up to a clay content of 0.6 vol %. With a higher amount of K-Hect, *G*_Ic_ decreased again. A similar trend was detected for the laminates with an MMT toughened matrix, but with a lower increase of *G*_Ic_ than for K-Hect. The fracture mechanisms shown by the SEM pictures in Figure 8 are comparable with the observations of the corresponding resin samples. The one without clay shows a brittle fracture behavior with only little shear yielding on a quite smooth fracture surface. The clay particles led to an elongation of the crack propagation path and therefore to an increase in fracture energy. While the fracture of CFRP happened more or less in one plane with MMT particles, the large K-Hect particles were able to initiate crack deflection into different crack planes. Additionally, K-Hect causes toughening by particle pull-out.

**Figure 9:**
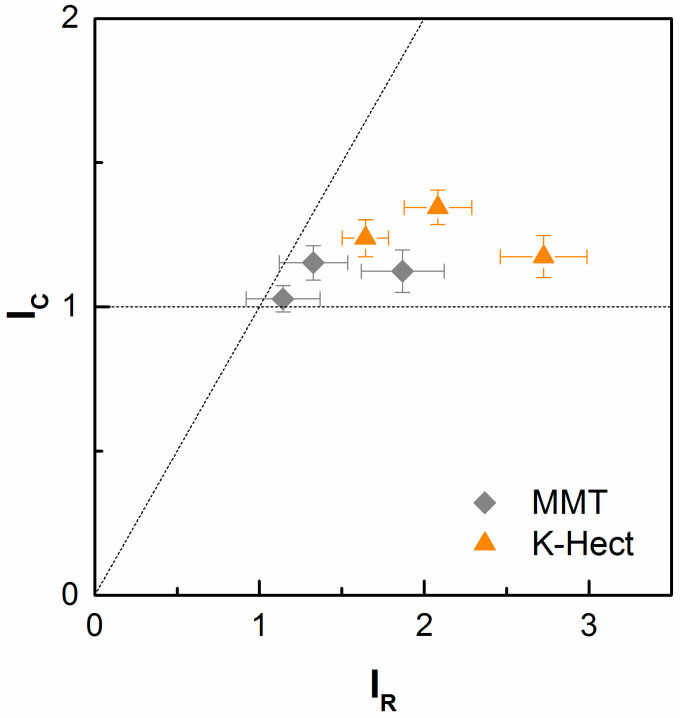
Comparison of improvement of fracture toughness by layered silicates in neat resin (*I*_R_) and composites (*I*_C_).

In accordance with the literature [6,32], a limitation of toughness improvement in CFRP in comparison to neat resin can be detected for both MMT and K-Hect particles at higher filler contents. Even if the increase in energy release rate of resin, given by Equation (5), almost reached a factor of 3, the improvement in CFRP, given by Equation (6), does not exceed a factor of 1.5 (Figure 9):

(5) $$I_{R}=\frac{G_{\mathrm{Ic}}\;of\;resin\;with\;layered\;silicates}{G_{\mathrm{Ic}}\;of\;neat\;resin}$$

(6) $$I_{C}=\frac{G_{\mathrm{Ic}}\;of\;laminate\;with\;layered\;silicates}{G_{\mathrm{Ic}}\;of\;laminate\;without\;layered\;silicates}$$

A possible reason for this behavior is the limitation of the extension of the plastic zone size in fiber composites. In the case of the resin specimens, an increase in the clay content leads to an increase in the plastic zone size. This corresponds directly to a higher fracture toughness of the resin. In contrast, the size of the plastic zone in laminates is limited by the fiber distance. An increase of particle content only increases the interlaminar toughness as long as the size of the plastic zone is smaller than the fiber distance. An *I*_R_ in the range from 1.0 to 1.5 can also be seen in research of Becker et al. [31], who investigated a material similar to the material in this study (UD carbon-fiber reinforced, fiber volume content of 49%, up to 7.5% organoclay). Significantly higher *I*_R_ could only be reached by Siddiqui et al. [27], but their material only had a fiber volume content of 20%. Therefore, the elongation of the crack propagation path is barely restricted by fibers.

**Figure 10:**
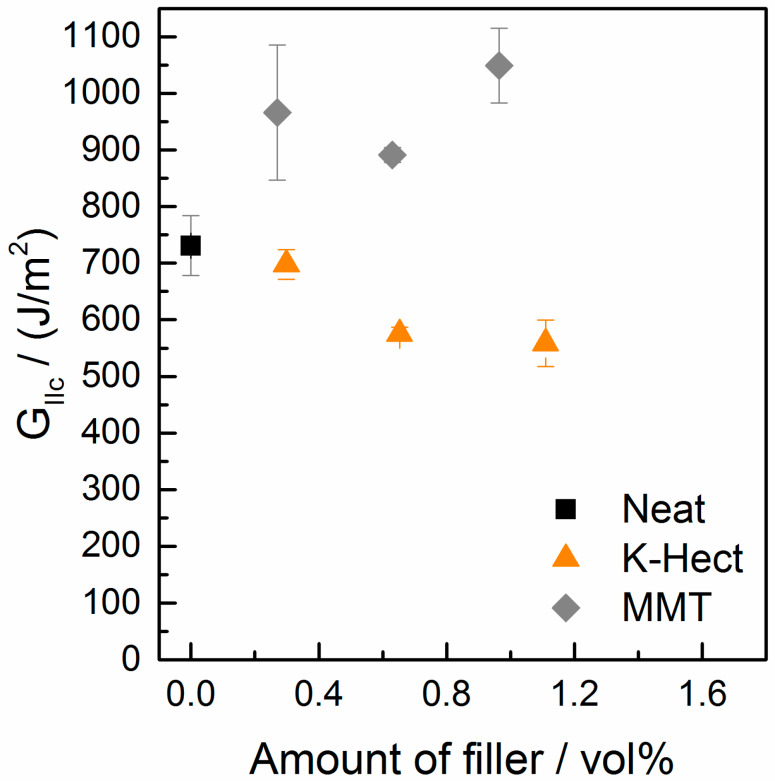
Energy release rate of mode II of laminates with different amounts of MMT or K-Hect.

**Figure 11:**
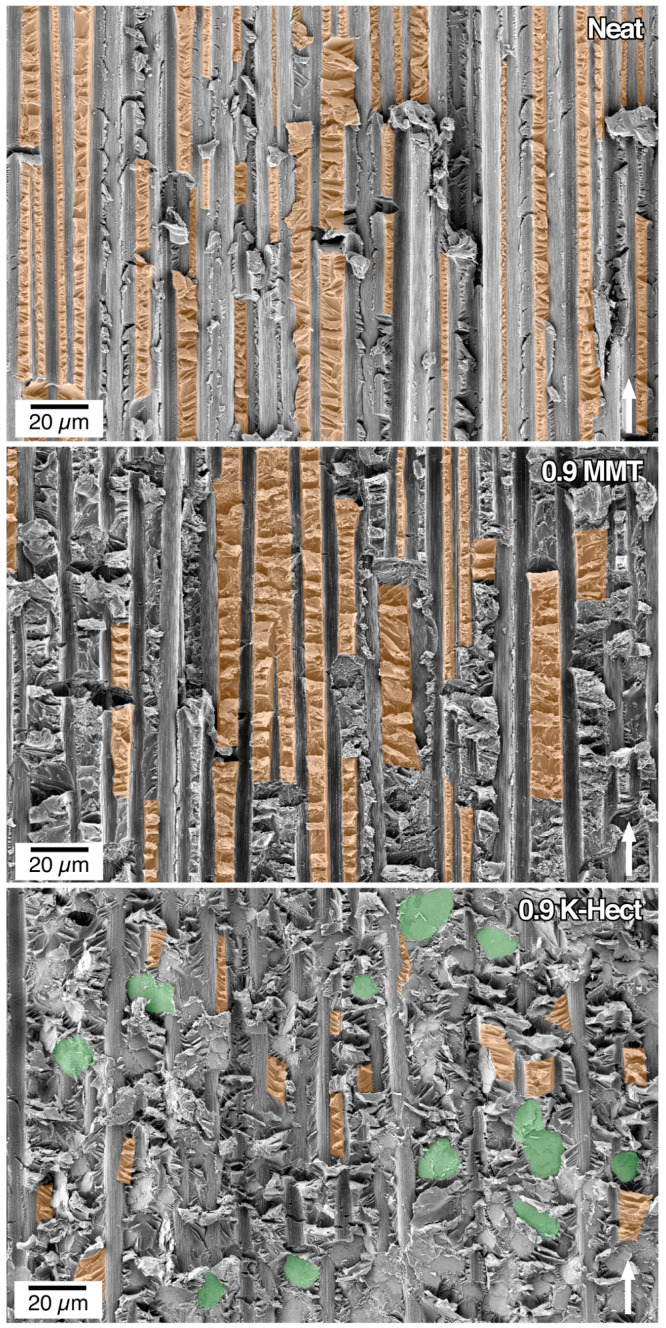
SEM pictures of fracture surface of end-notched-flexure samples (filler content in vol % is given in pictures). The white arrow shows the direction of crack propagation. Orange: hackling regions, green: K-Hect particles.

For *G*_IIc_, a significantly different behavior from *G*_Ic_ was detected (Figure 10). While the energy release rate increased by up to 43% with increasing MMT content, it decreased with growing K-Hect content. The combination of increasing values of *G*_Ic_ and *G*_IIc_ for MMT-reinforced composites is in contrast to literature, where at least one of both values decreases through addition of layered silicates [33,34]. It is also remarkable that there were major differences in mode II behavior with the two different layered silicates, while similar trends were detected in mode I behavior. The results of the mode I testing were based on the different fracture and toughening mechanisms mentioned before. As hackling is the dominating mechanism of the mode II fracture behavior, the hackle structures are discussed in detail in Figure 11. The orange marks show the regions where hackling structures could successfully be built up. A large hackling area is a sign of high *G*_IIc_ value. The fracture surface of the sample without layered silicates looks quite homogenous with regular hackle structures with an average width of 4.4 µm. The area covered with hackles was calculated from the SEM image to 28%. The image with MMT shows an area of hackling in the same magnitude (21%), but the width of the hackles of 6.5 µm is slightly higher. It can be concluded, that the MMT particles are placed inside the hackles and therefore reinforce these structures. This results in an increase of *G*_IIc_. In contrast, there was only a small area of 4%, where hackling was visible with K-Hect. The SEM picture also illustrates the reason for the low hackling amount. The areas marked in green are K-Hect particles that are oriented parallel to the fiber plies, and, therefore, exactly in the plane of crack propagation. This leads to an easier crack propagation along the particles and to areas where no hackling can occur, because the large layered silicates would hinder that. By these observations it can be concluded that there is a critical upper size for particles to “fit” into the hackling structures and reinforce them. As the average size of the hackling zones without particles is 4.4 µm, the small MMT particles with 400 nm can arrange within the zone, while K-Hect with 6 µm is too large.

**Figure 12:**
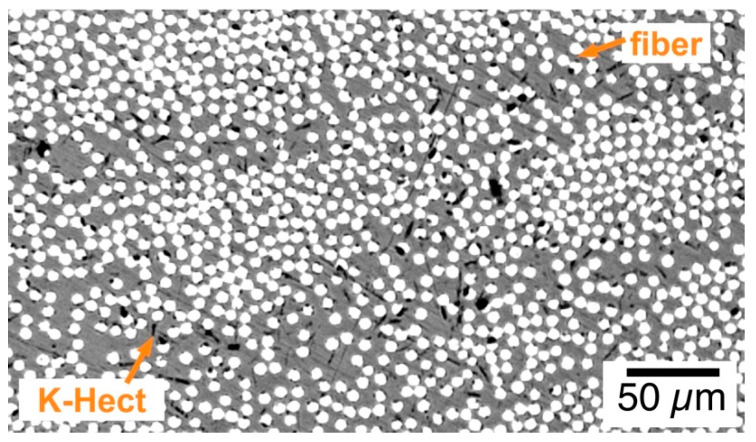
Microscopic picture of a cross-section of laminate with 0.6 vol % K-Hect. White: fibers; black: layered silicates; grey: epoxy matrix.

The reason for the orientation of particles can be explained with the help of Figure 12. This microscopy picture shows a cutoff of the cross-section of the laminate with 0.6 vol % of K-Hect. It can be seen that there are regions of tightly packed fiber bundles where K-Hect particles cannot be found because of their large size. They only occur in regions with a low amount of fibers. It can be concluded that these particles will not penetrate fiber bundles during prepreg production. Therefore, a high amount of particles will stay at the bottom of the prepreg below the fibers (because fibers are introduced from above). Also, during the autoclave process, the large K-Hect will remain between the fiber plies. Therefore, the actual particle concentration in this section was higher than expected. This may also be the reason for the decrease of *G*_Ic_ with an actually low amount of 1.1 vol % K-Hect, as in reality a very high number of particles hinder plastic deformation in a small resin rich zone. Another aspect that should be mentioned is the influence of fiber bridging. Extensive fiber bridging can increase the energy release rate *G*_Ic_. As shown in Figure 11, K-Hect is oriented between fiber layers. Therefore, fiber bridging is reduced, which leads to a decrease of *G*_Ic_. In summary, it can be said that the orientation of the K-Hect particles influences both *G*_Ic_ and *G*_IIc_, but is much more critical for mode II loading. MMT particles are much smaller than K-Hect, and, therefore, can be homogenously distributed between the fibers. For this reason, shear stress in mode II loading can homogenously be distributed and hackling structures can effectively be reinforced.

## 4. Conclusions

This work has investigated the influence of layered silicates on the interlaminar-fracture toughness of carbon-fiber-reinforced epoxies and especially the influence of particle size. Therefore, a synthetic layered silicate with a lateral extension of 6 µm and a natural MMT with a lateral extension of 400 nm were used.

It was shown that both particle types led to an improvement of *G*_Ic_. This was more pronounced for K-Hect, because additional toughening mechanisms like crack bridging, particle pull-out or crack deflection are enabled by large particle sizes. On the other hand, *G*_IIc_ can only be improved with MMT, whereas for K-Hect a decrease was seen. The reasons for these results are the differences in the position and orientation of particles within the laminate. MMT is small enough to penetrate fiber bundles and fit into hackling structures. Therefore, a homogenous stress distribution and a reinforcement of the hackling structures cause an increase in *G*_IIc_. In contrast, the K-Hect particles accumulate between the fiber(s) plies because their large size prohibits movements through the fiber bundles. Additionally, there is an orientation of the particles parallel to the fibers. While toughening mechanisms are still active under mode II loading, hackling is hindered in mode II, leading to a decrease of *G*_IIc_.

In conclusion, the study has shown that the larger particles are favorable for mode I fracture behavior, while the smaller particles show better performance in mode II. As there was a quite large difference between the sizes of both particle groups, it would be interesting to investigate the influence of particles with sizes in between the two types of this study. They could provide the possibility to combine the pronounced toughening mechanisms in mode I with an efficient interplay with hackling structures in mode II.
